# Sustaining the Activation of EGFR Signal by Inflammatory Cytokine IL17A Prompts Cell Proliferation and EGFR-TKI Resistance in Lung Cancer

**DOI:** 10.3390/cancers15133288

**Published:** 2023-06-22

**Authors:** Kai-Ling Lee, Tsung-Ching Lai, Wei-Jiunn Lee, Yu-Chieh Chen, Kuo-Hao Ho, Wen-Yueh Hung, Yi-Chieh Yang, Ming-Hsien Chan, Feng-Koo Hsieh, Chi-Li Chung, Jer-Hwa Chang, Ming-Hsien Chien

**Affiliations:** 1 Graduate Institute of Clinical Medicine, College of Medicine, Taipei Medical University, Taipei 11031, Taiwan; d118107005@tmu.edu.tw (K.-L.L.); wjlee@tmu.edu.tw (W.-J.L.); 111012@w.tmu.edu.tw (Y.-C.C.); d119106001@tmu.edu.tw (K.-H.H.); hungwenyueh@yahoo.com.tw (W.-Y.H.); rafiyang@tmu.edu.tw (Y.-C.Y.); 2 Division of Pulmonary Medicine, Department of Internal Medicine, Taipei Medical University Hospital, Taipei 110301, Taiwan; clchung@tmu.edu.tw; 3 Division of Pulmonary Medicine, Department of Internal Medicine, Wan Fang Hospital, Taipei Medical University, Taipei 11696, Taiwan; 109053@w.tmu.edu.tw; 4 Pulmonary Research Center, Wan Fang Hospital, Taipei Medical University, Taipei 11696, Taiwan; 5 Department of Medical Education and Research, Wan Fang Hospital, Taipei Medical University, Taipei 11696, Taiwan; 6 Department of Urology, School of Medicine, College of Medicine, Taipei Medical University, Taipei 11031, Taiwan; 7 Department of Medical Research, Tungs’ Taichung MetroHarbor Hospital, Taichung 433402, Taiwan; 8 Department of Biomedical Imaging and Radiological Sciences, National Yang Ming Chiao Tung University, Taipei 112304, Taiwan; mhchan@nycu.edu.tw; 9 The Genome Engineering & Stem Cell Center, School of Medicine, Washington University, St. Louis, MO 63130, USA; fhsieh@wustl.edu; 10 School of Respiratory Therapy, College of Medicine, Taipei Medical University, Taipei 11031, Taiwan; 11 Traditional Herbal Medicine Research Center, Taipei Medical University Hospital, Taipei 110301, Taiwan; 12 TMU Research Center of Cancer Translational Medicine, Taipei Medical University, Taipei 11031, Taiwan

**Keywords:** interleukin-17A, interleukin-17 receptor C, epidermal growth factor receptor, proliferation, non-small-cell lung cancer

## Abstract

**Simple Summary:**

Non-small-cell lung cancer (NSCLC) is a typical inflammation-associated cancer and epidermal growth factor (EGF) receptor (EGFR) mutations are the most common driver mutations of NSCLC. The aim of our study was to evaluate the impacts of the proinflammatory cytokine, interleukin (IL)-17A, on EGFR-mediated progression of NSCLC. In NSCLC cells with mutant EGFR, we found that the IL-17A/IL-17 receptor C (IL-17RC) axis enhanced phosphorylation of EGFR and Met, thereby promoting proliferation and resistance to EGFR-tyrosine kinase inhibitors (TKIs). In NSCLC cells with wild-type (WT) EGFR, we found that the IL-17A/IL-17RC axis enhanced EGF-induced EGFR activation and cell proliferation through causing an impairment of EGF-induced EGFR lysosomal degradation. Our results indicated that developing therapeutic strategies against IL-17A/IL-17RC would be valuable for NSCLC treatment.

**Abstract:**

Non-small-cell lung cancer (NSCLC) is a typical inflammation-associated cancer, and lung adenocarcinoma (LUAD) is the most common pathological subtype. Epidermal growth factor (EGF) receptor (EGFR) mutations are the most common driver mutations of LUAD, and they have been identified as important therapeutic targets by EGFR-tyrosine kinase inhibitors (TKIs). The proinflammatory cytokine, interleukin (IL)-17A, and IL-17A-producing cells were reported to be elevated in the tumor microenvironment and peripheral blood of NSCLC patients and to be correlated with tumor progression and poor prognoses. However, the pathophysiological role of IL-17A in NSCLC remains unclear, although some studies suggested its involvement in cancer cell invasion and metastasis. Herein, we observed that expressions of IL-17A and its receptor, IL-17 receptor C (IL-17RC), were elevated in LUAD tissues and were correlated with poor survival in different lung cancer cohorts. In LUAD cells with mutant EGFR, the IL-17A/IL-17RC axis was shown to enhance phosphorylation of EGFR and Met, thereby promoting proliferation and resistance to EGFR-TKIs such as afatinib. In LUAD cells with wild-type (WT) EGFR, we found that the IL-17A/IL-17RC axis enhanced EGF-induced EGFR activation and cell proliferation through causing impairment of EGF-induced EGFR lysosomal degradation. Collectively, our results indicated diverse impacts of the IL-17A/IL-17RC axis on EGFR activation in LUAD cells with WT and mutant EGFR and suggested that developing therapeutic strategies against IL-17A/IL-17RC would be valuable for LUAD treatment.

## 1. Introduction

The link between inflammation and tumorigenesis is well known, and studies have implicated many inflammatory components, such as interleukin (IL)-6, transforming growth factor (TGF)-β, IL-10, IL-33, etc., as key players in tumor development, growth, and metastasis [1]. IL-17 is one of the best-known proinflammatory cytokines found in the tumor microenvironment (TME) that mediates tumor progression [2]. IL-17A is the best-studied member of the IL-17 cytokine family which consists of six members (IL-17A~F), mainly secreted by a cluster of differentiation 4-positive (CD4^+^) T helper (Th) lymphocytes cells (Th17), CD8^+^ T cells, γδ T cells, and natural killer (NK) cells [3,4]. In addition to immune cells, some studies have suggested that tumor cells themselves might acquire the ability to produce IL-17A under certain conditions or in specific types of tumors. For example, colorectal and breast cancers are capable of producing IL-17 to guide IL-17R-positive neutrophils’ directional migration [5]. IL-17A targets various cells which contain its receptor, composed of IL-17RA and IL-17RC dimers, which results in host defense, chronic inflammation, tissue destruction and repair, and oncogenesis [6,7]. IL-17A and its receptors are expressed across different tumor types and contribute to a protumor niche via different pathways, such as promoting tumor chemoresistance and proliferation through extracellular signal-regulated kinase 1/2 (ERK1/2) in breast cancer [8]. IL-17A promotes tumor immunosuppression and induces immune escape in colorectal cancer [9]. IL-17A was shown to stimulate vascular endothelial growth factor A (VEGFA) production and promote angiogenesis in gastric [10] and colorectal cancers [11]. In non-solid tumors, IL-17A was also reported to increase multiple myeloma cell viability via upregulating spleen tyrosine kinase [12].

Lung cancer is known to be a typical inflammation-associated cancer [13]. Non-small-cell lung cancer (NSCLC) accounts for 85% of total lung cancers, and lung adenocarcinoma (LUAD) is the most common histological subtype of NSCLC [14]. Epidermal growth factor (EGF) receptor (EGFR) mutations are the most common driver mutations of LUAD that promote survival and proliferation of cancer cells, and EGFR-tyrosine kinase inhibitor (TKI) therapies are effective in this subset of LUAD cases, but acquired resistance to these TKIs is inevitable [15,16]. In contrast to mutant EGFR, EGFR-TKIs show minimal effects on tumors with wild-type (WT) EGFR, and EGFR WT allele amplification was recently shown to contribute to acquired resistance to EGFR-TKIs [17]. Previous reports indicated that IL-17A can induce activation of EGFR to promote wound healing and skin tumorigenesis [18]. In breast cancer, IL-17E was synergized with EGF and conferred resistance to EGFR-TKIs [19]. Indeed, elevated levels of IL-17A-producing cells such as Th17, IL-17A, and its receptor were observed in NSCLC specimens and were correlated with tumor progression and poor prognoses [20,21,22,23]. For example, IL-17A was reported to induce invasion/metastasis of NSCLC cells via inducing epithelial–mesenchymal transition and upregulation of matrix metallopeptidase (MMP)-2 and MMP-9 [20,21]. However, the crosstalk between IL-17A and EGFR in LUAD remains unknown. This study attempted to determine the impacts of IL-17A on EGFR-mediated tumor proliferation and responses to EGFR-TKI treatment in LUAD, and to further investigate the mechanisms involved in the crosstalk between IL-17A and EGFR signaling.

## 2. Materials and Methods

### 2.1. Data Collection from Bioinformatics Analyses

The UALCAN online database (http://ualcan.path.uab.edu (accessed on 24 June 2022) [24] was used to calculate *IL-17A* and *IL-17RC* gene expression levels and clinicopathologic parameters such as clinical stage and smoking status in The Cancer Genome Atlas (TCGA) dataset in patients with NSCLC, especially those with LUAD. The Kaplan–Meier (KM) plotter database (https://kmplot.com/analysis/ (accessed on 27 June 2022) was used to analyze the effects of IL-17A, IL-17RA, and IL-17RC levels on the survival of NSCLC subjects which were obtained from Gene Expression Omnibus (GEO) and TCGA datasets. Patients were divided into two groups including high and low expressions of IL-17A, IL-17RA, or IL-17RC, based on the best cutoff values of gene expression.

### 2.2. Cell Lines and Cell Culture

Human NSCLC cell lines including A549 and H23 (wild-type EGFR), H1975, HCC827, and PC9 (mutant EGFR) were purchased from American Type Culture Collection (ATCC, Manassas, VA, USA). Cells were cultured in RPMI 1640 medium (Gibco, Grand Island, NY, USA) supplemented with 10% fetal bovine serum (FBS), 100 U/mL penicillin, and 100 µg/mL streptomycin (Life Technologies, Gaithersburg, MD, USA) at 37 °C in a humidified atmosphere containing 5% CO_2_.

### 2.3. Reagents, Chemical Inhibitors, and Antibodies

The human recombinant IL-17A (rhIL-17A, cyt-250, and #Q16552) was purchased from Prospec (Ness-Ziona, Israel) and R&D Systems (Minneapolis, MN, USA). The human recombinant EGF (10605-HNAE) was obtained from Sino Biological (Beijing, China). Afatinib was purchased from Sigma Chemical (St. Louis, MO, USA). Antibodies used in the Western blot, flow cytometric, and immunofluorescence (IF) analyses are shown as follows: antibodies for α-tubulin (#2144) and phosphorylated (p-) and unphosphorylated forms of EGFR (#3777 and #4267), Met (#3077 and #8198), AKT (#9271 and #9272), extracellular signal-regulated kinase (ERK; #4370 and #4695), and Src (#2101) were all acquired from Cell Signaling Technology (Danvers, MA, USA). Santa Cruz Biotechnology (Santa Cruz, CA, USA) provided antibodies for IL-17A (sc-374218), EGFR (sc-101), lysosomal-associated membrane protein 1 (LAMP1; sc-20011), and GAPDH (sc-32233), while Proteintech Group (Chicago, IL, USA) supplied the antibody for His-tag (66005-1-ig). A fluorescent AF594-labeled donkey anti-mouse antibody (A-21203) and AF488-labeled goat anti-rabbit antibody (A-11034) were obtained from Invitrogen (Waltham, MA, USA). Unless otherwise specified, other chemicals used in this study were purchased from Sigma Chemical.

### 2.4. DNA Transient Transfection

The pENTER-IL17A plasmid was provided by Dr. Han-Pin Kuo (Taipei Medical University, Taiwan). To overexpress IL-17A, 3 µg of plasmid was transfected into 10^6^ cells cultured on a 6 cm Petri dish using the Lipofectamine 3000 transfection reagent protocol (Invitrogen, Carlsbad, CA, USA). After 6 h of transfection, the culture medium was replaced with complete medium. At 24 h after transfection, cells were analyzed for expression and secretion of IL-17A, and proliferation.

### 2.5. Western Blot Assay

Protein lysates were prepared, and sodium dodecylsulfate polyacrylamide gel electrophoresis (SDS-PAGE) and blotting were conducted using a Bio-Rad system (Bio-Rad Laboratories, Hercules, CA, USA) according to a previously described protocol [25]. The indicated primary antibodies were diluted 1:1000 and incubated with blots overnight at 4 °C. After incubation with peroxidase-labeled secondary antibodies, the signal was enhanced using a Chemiluminescent Substrate Kit (T-Pro Biotechnology, New Taipei City, Taiwan) and measured with an image recording system (CLUBIO, Taipei, Taiwan). Image-Pro Plus software, V6.0 (Media Cybernetics, Silver Spring, MD, USA) was used to quantify the density of specific bands.

### 2.6. Dot Blot Assay

After transfecting the pENTER-IL17A plasmid into NSCLC cells for 24 h, the cell culture medium was replaced with serum-free medium for another 24 h and then harvested for dot blotting. After a nitrocellulose membrane (Amersham Biosciences, Amersham, UK) was transferred into the dot blot chamber, 300 µL of medium was loaded into each well and aspirated very slowly through the membrane by a suction pump. Hybridization of the antibody and detection of signals were performed according to the Western blot protocol.

### 2.7. Cell Proliferation Assay

A549, PC9, and HCC827 NSCLC cells (3 × 10^3^) were seeded in 96-well plates, received different treatment protocols for 24 and 48 h, and then were subjected to a cell proliferation assay (Cell Counting Kit-8, CCK8 assay; Sigma-Aldrich) according to the manufacturer’s instructions. Data were collected from three replicates.

### 2.8. Lentiviral Infection

Lentiviral constructs expressing IL-17RC-specific short hairpin (sh)RNAs were purchased from the RNA Technology Platform and Gene Manipulation Core Facility at Academic Sinica (Taipei, Taiwan). The preparation of lentiviral particles and infection of lentiviral particles expressing control or IL-17RC shRNAs with NSCLC cells were established according to a previously described protocol [26]. Briefly, we seeded 5 × 10^5^ cells on 6 cm Petri dishes and infected cells with a lentivirus at a multiplicity of infection (MOI) of 5. After 3 days, 1 µg/mL puromycin was applied to select infected cells. The target sequence of IL-17RC shRNA was 5′-CGCCTTGGAGAGTACTTACTA-3′.

### 2.9. Human Phospho-Receptor Tyrosine Kinase (RTK) and Phospho-Kinase Arrays

Experiments were conducted following the manual of the Human Phospho-RTK array kit (ARY001B; R&D Systems) and Human Phospho-Kinase Array kit (ARY003B; R&D Systems). For the Phospho-RTK array, PC9 cells were treated with the vehicle or rhIL-17A for 10 min. For the Phospho-Kinase array, serum-starved A549 cells were treated with the vehicle, EGF (100 ng/mL) alone, or EGF combined with rhIL-17A for 10 min. Total protein lysates were harvested with lysis buffer provided by the kits, and 200 µg protein was applied to the membrane containing different indicated antibodies for the binding reaction. After reacting with specific antibodies and target proteins, signals were detected using a chemiluminescent horseradish peroxidase (HRP) substrate and continuously recorded by an image recording system (CLUBIO). Spot densities were normalized against respective reference array spots and then against the controls.

### 2.10. Flow Cytometric Analysis

Serum-starved A549 cells were treated with vehicle, EGF (100 ng/mL) alone, or EGF combined with rhIL-17A for 10 and 30 min. Cells were collected and then incubated with an EGFR antibody (sc-101, 1:100) on ice. After washing cells with phosphate-buffered saline (PBS), a fluorescent secondary antibody was added. Fluorescent signals of cells were then detected using FASC cytometry (Beckman, Indianapolis, IN, USA) and analyzed with CytExpert software (V2.0.0.153, Beckman).

### 2.11. IF and Confocal Microscopic Analysis

Serum-starved A549 cells were treated with vehicle, EGF (100 ng/mL) alone, or EGF combined with rhIL-17A (10 ng/mL) for 30 min before being fixed with paraformaldehyde for 30 min at room temperature (RT). Cells were then blocked with 5% bovine serum albumin (BSA) and incubated with anti-EGFR (#4267, 1:50) and anti-LAMP1 (sc20011, 1:20) antibodies for 1 h at RT. After washing, cells were incubated with an AF488-labeled goat anti-rabbit antibody (1:1000) and AF594-labeled donkey anti-mouse antibody (1:1000) for 30 min at RT. The sample was finally stained with DAPI for 5 min and mounted with mounting medium for confocal microscopic imaging. Images were analyzed using MetaMorph software, V7.8.4.0 (Molecular Devices, San Jose, CA, USA).

### 2.12. Statistical Analysis

All values are presented as the mean ± standard deviation (SD), and we used the Statistical Package for Social Science software, vers. 18 (SPSS, Chicago, IL, USA) and GraphPad Prism 7 (GraphPad Software, San Diego, CA, USA) to analyze the statistical significance of our results. Student’s *t*-test was used to conduct the statistical analyses, and significance was accepted at *p* < 0.05.

## 3. Results

### 3.1. Expression Levels of IL-17A and IL-17RC and Their Prognostic Potential in NSCLC

**Figure 1:**
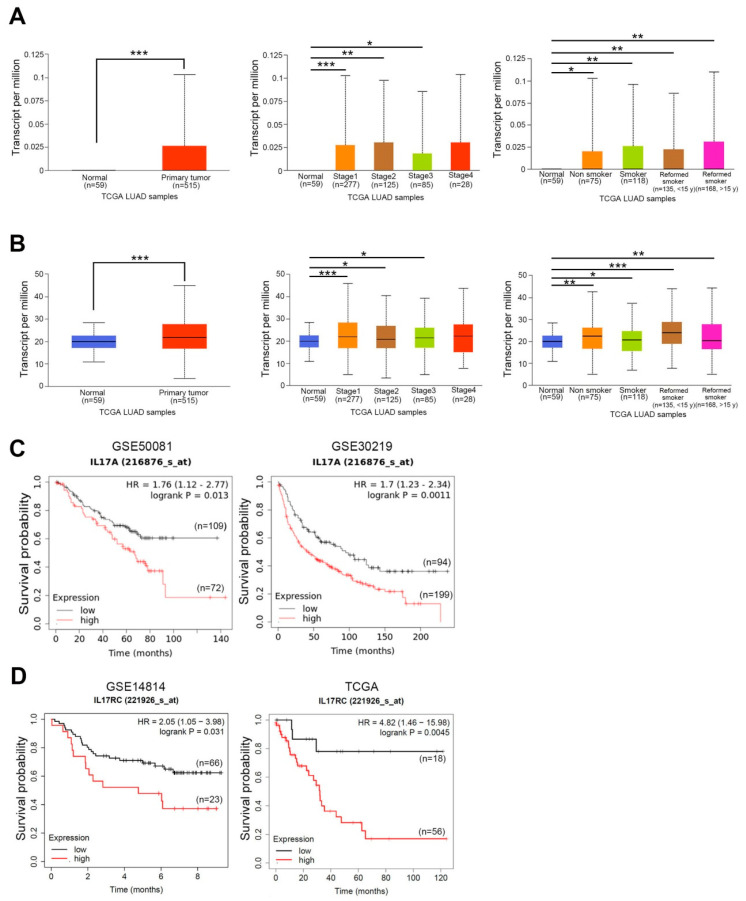
Clinical significance of interleukin (IL)-17A and its receptor in patients with non-small-cell lung cancer (NSCLC). (**A**,**B**) UALCAN portal analysis of NSCLC samples (lung adenocarcinoma, LUAD) from TCGA database. Comparison of IL-17A (**A**, **left panel**) and IL-17 receptor C (IL-17RC) (**B**, **left panel**) expressions between normal and tumor tissues. Expressions of IL-17A (**A**, **middle panel**) and IL-17RC (**B**, **middle panel**) in tumor tissues obtained from NSCLC patients at different clinical stages. Expressions of IL-17A (**A**, **right panel**) and IL-17RC (**B**, **right panel**) in tumor tissues obtained from NSCLC patients with or without a smoking habit. * *p* < 0.05, ** *p* < 0.01, *** *p* < 0.001. (**C**,**D**) Correlation between IL-17A (**C**) and IL-17RC (**D**) expressions with overall survival (OS) in patients with lung cancer as determined using a Kaplan–Meier plotter (KM) plotter database. Gene expressions were dichotomized into high and low values using the best cutoff value. *p* < 0.05 was considered to indicate a statistically significant difference. HR, hazard ratio.

We first evaluated differences in expressions of IL-17A and its receptor, IL-17RC, between tumors and normal tissues in NSCLC using the UALCAN database [24]. According to RNA sequencing data from TCGA, significantly higher IL-17A and IL-17RC transcripts were observed in tumors compared to normal tissues (*p* < 0.001) (Figure 1A,B, left panel). We further performed a subgroup analysis of multiple clinical pathological features of NSCLC in the UALCAN database. We also observed that IL-17A and IL-17RC expression increased in NSCLC stages 1 to 3 compared to normal samples (Figure 1A,B, middle panel). Furthermore, the smoking status did not significantly influence expression levels, as IL-17A and IL-17RC expressions were significantly upregulated in NSCLC compared to those in normal tissues, irrespective of the smoking history (Figure 1A,B, right panel). Correlations between IL-17A or its receptors with the overall survival (OS) of NSCLC patients were analyzed using the KM-plotter, and the test populations were obtained from GEO or TCGA datasets. As shown in Figure 1C,D, a shorter OS time was found for NSCLC patients with high IL-17A or IL-17RC expression compared to patients with low IL-17A or IL-17RC expression. In addition to IL-17RC, another receptor of IL-17A, IL-17RA, also showed a poor prognostic effect on patients with NSCLC (Figure S1). Overall, these clinical data suggest that the IL-17A-IL17-RA/IL-17RC axis may play a critical role in modulating NSCLC development.

### 3.2. IL-17A Induces Cell Proliferation of NSCLC Cells with Wild-Type (WT) or Mutant EGFR

**Figure 2:**
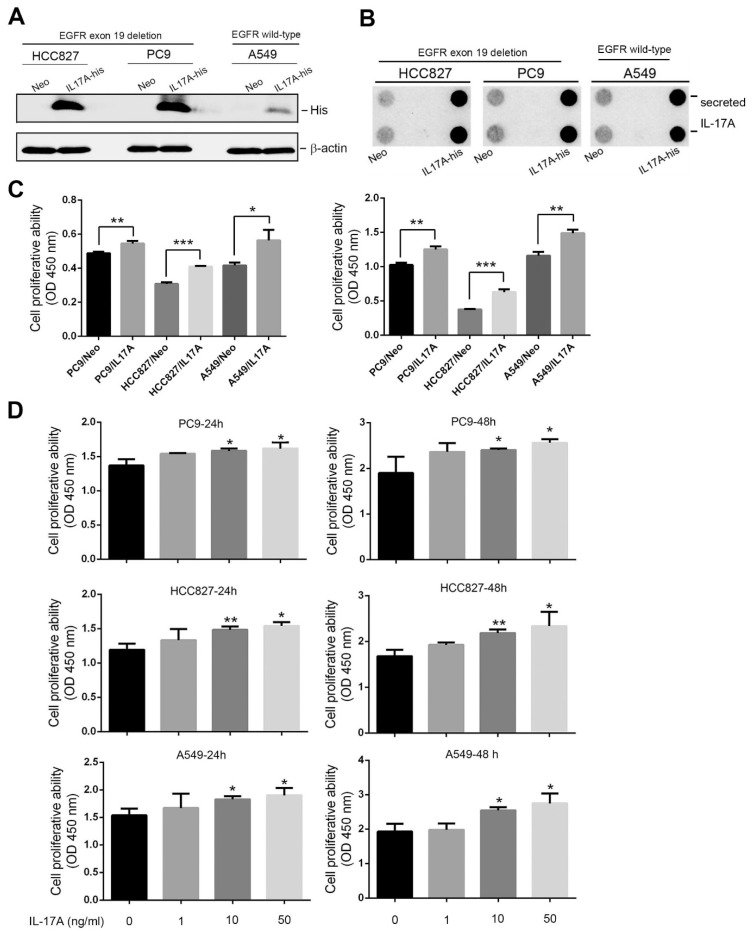
Interleukin (IL)-17A induces proliferation of human non-small-cell lung cancer (NSCLC) cells harboring wild-type (WT) or mutant epidermal growth factor receptor (EGFR). (**A**) Western blot analysis of IL-17A expressions in PC9, HCC827, and A549 cells after transfecting a control vector (Neo) or IL-17A-expressing vector (IL-17A-His). (**B**) Dot blot analysis of secreted IL-17A using conditioned media of IL-17A-His-transfected PC9, HCC 827, and A549 cells. The uncropped blots are shown in the Supplemental Materials. (**C**) Proliferation rates of IL-17A-overexpressing PC9, HCC827, and A549 cells were measured by performing a CCK8 assay. Proliferation rates had significantly increased in IL-17A-His-transfected NSCLC cells after 24 (left) and 48 h (right) compared to control cells. (**D**) PC9, HCC827, and A549 cells were treated with 0, 1, 10, or 50 ng/mL recombinant human (rh)IL-17A for 24 and 48 h. The effect of rhIL-17A on proliferation of these NSCLC cells was evaluated by a CCK-8 assay. * *p* < 0.05, ** *p* < 0.01, *** *p* < 0.001 compared to the control group.

Next, we investigated the effects of IL-17A locally produced by tumors as a means to evaluate its biologic function. We first overexpressed His-tagged IL-17A in three NSCLC cell lines which, respectively, harbor WT (A549) and mutant EGFR (PC9 and HCC827, exon 19 deletion) (Figure 2A). Increased secretion of IL-17A was also observed in these three NSCLC cells (Figure 2B). We next examined the effect of IL-17A on cell proliferation using the highly sensitive cell proliferation detection kit, CCK-8, and observed that proliferation rates of all IL-17A-overexpressing NSCLC cells increased compared to control cells after 24 (Figure 2C, left panel) and 48 h (Figure 2C, right panel) of incubation. We further treated A549, PC9, and HCC827 cells with different concentrations of rhIL-17A for 24 and 48 h. Results showed that cell proliferation rates of all tested cells were elevated after rhIL-17A treatment in concentration-dependent manners (Figure 2D). These results suggested that regardless of the overexpression of IL-17A by gene transduction or rhIL-17A treatment, all promoted the proliferation of NSCLC cells, irrespective of the EGFR status.

### 3.3. IL-17A Enhances EGFR and MET Activation and Confers In Vitro Resistance to EGFR-Targeted Therapies in NSCLC Cells with Mutant EGFR

**Figure 3:**
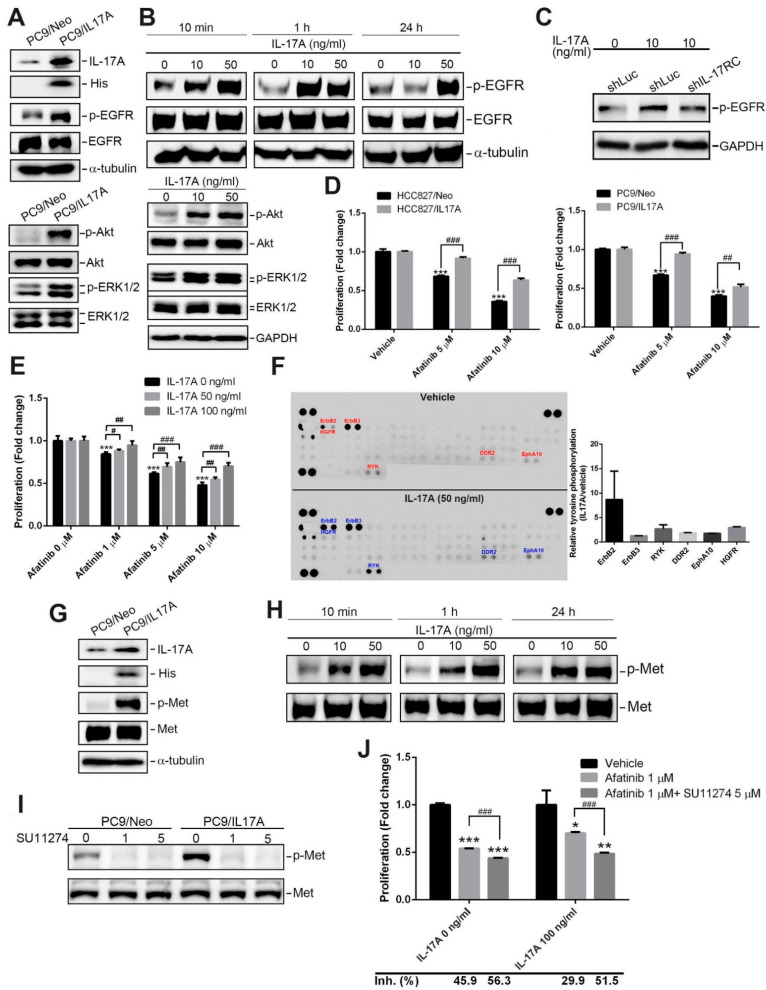
Interleukin (IL)-17A facilitates phosphorylation of epidermal growth factor receptor (EGFR) and Met and subsequently induces EGFR-tyrosine kinase inhibitor (TKI) resistance in human non-small-cell lung cancer (NSCLC) cells harboring mutant-EGFR. (**A**,**B**) Detection of phosphorylated (p)-EGFR and its downstream signaling by Western blotting after overexpression of IL-17A (**A**) and treatment with rhIL-17A at different concentrations and time points (**B**) in EGFR-mutant PC9 cells. (**C**) Western blot analysis of p-EGFR in PC9 cells after transducing shIL-17RC or control shRNA (shLuc) and treatment with rhIL-17A or the vehicle. Quantitative results of p-EGFR proteins were adjusted to GAPDH protein levels. (**D**) A CCK8 assay was performed to evaluate the proliferation inhibitory effect of afatinib treatment for 72 h in PC9 or HCC827 cells with or without IL-17A overexpression *** *p* < 0.001, ^##^ *p* < 0.01, ^###^ *p* < 0.001. (**E**) Proliferation rates of PC9 cells that received different concentrations of afatinib combined with or without rhIL-17A for 24 h. *** *p* < 0.001 compared to the control group. ^#^ *p* < 0.05, ^##^ *p* < 0.01, ^###^ *p* < 0.001 compared to the afatinib-treated only group. (**F**) Differential expression levels of phosphorylated receptor tyrosine kinases (RTKs) in PC9 cell lysates following 24 h treatment with rhIL-17A. An antibody array (R&D Systems) was used to detect 49 different phosphorylated human RTKs. The left panel shows representative array blots. The right panel shows a quantitative analysis of phosphorylated RTKs using a densitometer. Values are presented as the mean ± SD. *n* = 2. (**G**,**H**) Detection of phosphorylated (p)-Met by Western blotting after overexpression of IL-17A (**G**) and treatment with rhIL-17A at different concentrations and time points (**H**) in PC9 cells. (**I**) Detection of p-Met by Western blotting in PC9/IL-17A and PC9/Neo cells receiving different concentrations of SU11274. The uncropped blots are shown in the Supplemental Materials. (**J**) Proliferation rates of rhIL-17A-treated or vehicle-treated PC9 cells after receiving afatinib or afatinib+SU11274 for 48 h. * *p* < 0.05, ** *p* < 0.01, *** *p* < 0.001 compared to the control group. ^###^ *p* < 0.001 compared to the afatinib-treated only group.

EGFR mutations are the most common driver mutations of NSCLC which promote the survival and proliferation of cells [27]. IL-17E was reported to induce EGFR activation in breast cancer cells [19]. Herein, we observed that IL-17A overexpression increased the phosphorylation of EGFR (p-Y1068) and EGFR-driven signaling pathways including Akt and ERK in EGFR-mutant PC9 cells (Figure 3A). Enhancement of EGFR activation by IL-17A overexpression was also observed in other EGFR-mutant NSCLC cells, such as HCC827 cells, even though H1975 cells harbored double mutations of EGFR (L858R and T790M) (Figure S2). Moreover, treatment of PC9 and HCC827 cells with rhIL-17A also enhanced activation of EGFR and its downstream signals, Akt and ERK (Figure 3B and Figure S3). To further investigate whether the receptor of IL-17A is involved in IL-17A-induced EGFR activation, we tried to knock down IL-17RC by a lentiviral-based shRNA in PC9 cells (Figure S4A). We found that IL-17RC depletion abrogated the rhIL-17A-induced increase in EGFR phosphorylation (Figure 3C), suggesting that IL-17A-enhanced EGFR activation is dependent on IL-17RC. Resistance to EGFR-TKIs is often associated with sustained phosphorylation of EGFR [28]. Therefore, we further verified whether IL-17A can affect the therapeutic potential of EGFR-TKI against EGFR-mutant NSCLC cells. Afatinib is a second-generation EGFR-TKI, and also a pan-ErbB inhibitor, and inhibits cell proliferation in EGFR-mutant NSCLC cells [16]. We observed that afatinib treatment concentration-dependently suppressed the proliferation of PC9 and HCC827 cells, and the inhibitory effect of afatinib was significantly reversed by IL-17A overexpression (Figure 3D) or treatment with rhIL-17A (50 and 100 ng/mL) (Figure 3E). Moreover, we conducted additional experiments to assess the effect of rhIL-17A on the IC50 (half maximal inhibitory concentration) value of afatinib in PC9 cells after 24 h and 48 h of treatment. We observed that the IC50 of afatinib after 48 h of treatment was higher in cells treated with 100 ng/mL rhIL-17A compared to control cells (Figure S5). Taken together, these results suggest that the IL-17A/IL17RC axis contributes to afatinib resistance in EGFR-mutant NSCLC cells. To further dissect the signaling cascade induced by IL-17A that is responsible for afatinib resistance, we next performed proteomic screening with a human phospho-RTK array (Figure 3F, left panel). Among 49 phosphorylated RTKs, six RTKs including ErbB2, ErbB3, RYK, discoidin domain receptor 2 (DDR2), ephrin receptor A10 (EphA10), and hepatocyte growth factor receptor (HGFR)/c-Met, were all upregulated in rhIL-17A-treated PC9 cells compared to vehicle-treated cells (Figure 3F, right panel). Amplification and activation of c-Met were recognized as important bypass pathways involved in resistance to EGFR-TKIs [16]. Herein, a Western blot analysis was performed to validate that regardless of rhIL-17A treatment or IL-17A overexpression, both enhanced c-Met activation in PC9 and HCC827 cells (Figure 3G,H, Figures S2 and S3). Concomitant treatment of NSCLC cell lines with a MET inhibitor (SU11274) caused increased sensitivity to EGFR-TKIs, such as afatinib [29]. Herein, we observed that treatment of PC9 cells with SU11274 suppressed IL-17A-induced Met phosphorylation (Figure 3I). Cotreatment of afatinib with SU11274 enhanced afatinib-induced inhibition of cell proliferation and reversed IL-17A-caused afatinib resistance in PC9 cells (Figure 3J). Taken together, these results suggested that IL-17A activates compensatory EGFR signaling via phosphorylating c-Met under afatinib treatment in EGFR-mutant NSCLC cells.

### 3.4. IL-17A Enhances EGF-Induced EGFR Activation and Cell Proliferation through Causing Prevention of EGF-Mediated EGFR Degradation

**Figure 4:**
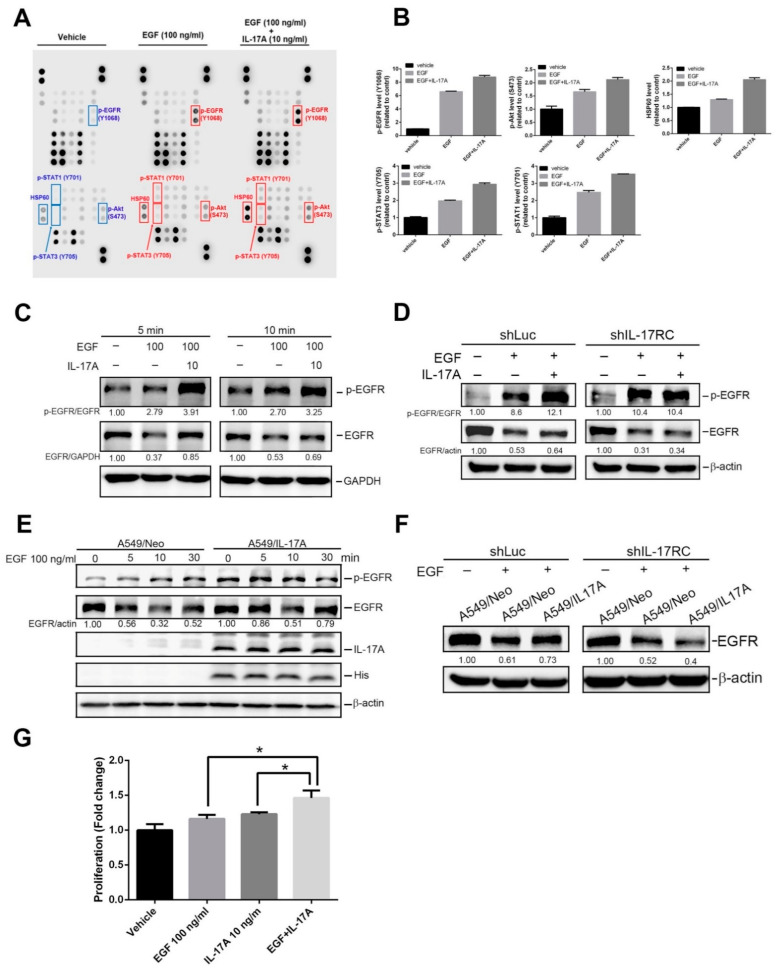
Interleukin (IL)-17A synergizes with epidermal growth factor (EGF) to trigger EGF receptor (EGFR) activation via preventing EGF-mediated EGFR degradation in human non-small-cell lung cancer (NSCLC) cells harboring wild-type (WT)-EGFR. (**A**,**B**) Differential expression levels of phosphorylated kinases in A549 cell lysates following 10 min treatment with EGF or EGF + rhIL-17A. An antibody array (R&D Systems) was used to detect 37 different phosphorylated kinases and two related total proteins. Representative array blots are shown in (**A**). Quantitative analysis of phosphorylated kinases or total proteins using a densitometer are shown in (**B**). (**C**) Detection of phosphorylated (p)-EGFR and total EGFR by Western blotting after treatment of A549 cells with EGF or EGF+rhIL-17A for the indicated time points. (**D**) Western blot analysis of p-EGFR and total EGFR in A549 cells after transducing shIL-17RC or control shRNA (shLuc) and treatment with EGF or EGF+rhIL-17A for 10 min. (**E**) IL-17A-His-transfected A549 or control cells were treated with EGF (100 ng/mL) for the indicated time points, and expression levels of p-EGFR and total EGFR were further evaluated by a Western blot analysis. (**F**) A549/shLuc and A549/shIL-17RC cells were transfected with IL-17A-His or a control vector and treated with EGF for 10 min, and total EGFR was further detected. Quantitative results of p-EGFR and total EGFR proteins from (**C**–**F**) were, respectively, adjusted to total EGFR and β-actin or GAPDH protein levels. The uncropped blots are shown in the Supplemental Materials. (**G**) A CCK8 assay was performed to evaluate proliferation rates of A549 cells which were treated with the vehicle, EGF (100 ng/mL), rhIL-17A (10 ng/mL), or EGF+rhIL-17A for 24 h. * *p* < 0.05, compared to the EGF- or rhIL-17A-treated only group.

We speculated that the effect of IL-17A on WT-EGFR NSCLC might be different from that on mutant-EGFR NSCLC as mutant-EGFR is constitutively activated but WT-EGFR is not. WT-EGFR NSCLC is dependent on the binding of the ligand, EGF, to trigger downstream signals and induce cell proliferation [30]. A previous study reported that IL-17E synergizes with EGF and confers EGFR-TKI resistance in breast cancer [19]. Herein, WT-EGFR A549 cells were treated with EGF with or without rhIL-17A, and cell lysates were applied to a human phospho-kinase (PPK) array (Figure 4A). As shown on the PPK density quantification (Figure 4B), higher activation of EGFR and of its downstream signaling kinase proteins, including signal transduction and activator of transcription 3 (STAT3) and Akt, was observed in cells under combined treatment with EGF and rhIL-17A than with EGF treatment alone. The enhancement of EGF-induced EGFR activation by rhIL-17A was further validated by a Western blot analysis in both WT-EGFR A549 and H23 NSCLC cells (Figure 4C and Figure S6), indicating that IL-17A also modulates activation of WT-EGFR in NSCLC cells. EGF is one of the ligands that activates EGFR signaling, but it also mediates degradation of EGFR [31]. Actually, total EGFR was downregulated in A549 and H23 cells treated with EGF for 5 or 10 min, and this phenomenon was reversed by cotreatment with rhIL-17A (Figure 4C and Figure S6). When we knocked down IL-17RC in A549 cells (Figure S4B), the enhanced EGFR activation and attenuated EGFR degradation achieved by IL-17A in EGF-treated cells were aborted (Figure 4D). Similar to rhIL-17A-treated cells, overexpression of IL-17A in A549 cells also promoted EGFR activation and prevented EGFR degradation caused by EGF (Figure 4E). Preventing EGF-induced EGFR degradation by IL-17A overexpression was also aborted when IL-17RC was depleted (Figure 4F). Taken together, our results suggested that the IL-17A-IL-17RC axis may enhance EGF-induced EGFR activation via preventing EGFR degradation. Functionally, we further evaluated the contribution of IL-17A to EGF-mediated cell growth and found that cotreatment of rhIL-17A with EGF enhanced the EGF-induced increase in cell proliferation in A549 cells (Figure 4G).

### 3.5. IL-17A Prevents EGF-Induced EGFR Downregulation through Causing Impairment of the Lysosomal Degradation of EGFR

**Figure 5:**
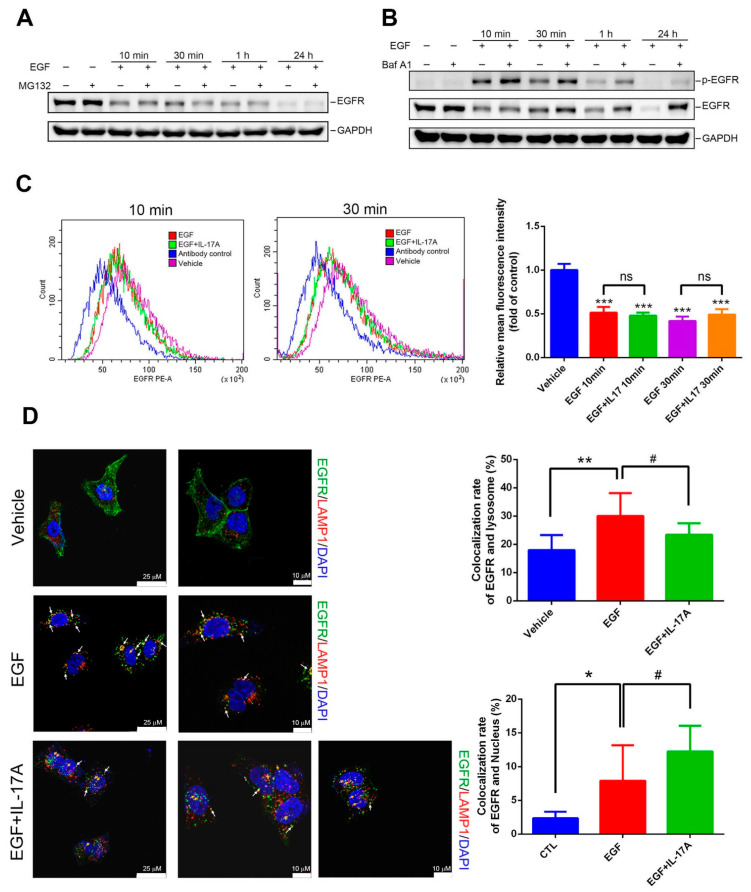
Interleukin (IL)-17A prevents epidermal growth factor (EGF)-mediated EGF receptor (EGFR) degradation via impairing the transport of EGFR to lysosomes in human non-small-cell lung cancer (NSCLC) cells harboring wild-type (WT)-EGFR. (**A**,**B**) Serum-starved A549 cells were pretreated with MG132 (20 µM) (**A**) or bafilomycin A1 (20 nM) (**B**) for 1 h, and then 100 ng/mL EGF was added for the indicated time points. Cell lysates from (**A**,**B**) were subjected to a Western blot analysis to detect phosphorylated (p)-EGFR and total EGFR expressions. The uncropped blots are shown in the Supplemental Materials. (**C**) Serum-starved A549 cells were treated with EGF (100 ng/mL) alone or EGF combined with rhIL-17A (10 ng/mL) for the indicated time points (10 and 30 min). Membrane EGFR levels were detected by a FASC analysis after staining with a mouse mAb to EGFR or mouse isotype control antibodies. The mean fluorescence intensity was a measure of the level of EGFR expression. *** *p* < 0.001 compared to the control group. (**D**) Different colocalization of lysosomal-associated membrane protein 1 (LAMP1) and endocytosed EGFR after 30 min of EGF or EGF+rhIL-17A treatment. Left panel: Cells were fixed, permeabilized, and stained with anti-EGFR (green), anti-LAMP1 (red), and DAPI (blue) for nuclear staining, and further examined by confocal microscopy. White arrows point to colocalization of EGFR and lysosomes. Orange arrows point to the nuclear translocation of EGFR. Right panel: Colocalization rate of EGFR and lysosome or nucleus was analyzed by MetaMorph software (version 7.8.4.0). * *p* < 0.05, ** *p* < 0.01 compared to the vehicle control group. ^#^ *p* < 0.05 compared to the EGF-treated alone group. ns: No Significance.

Both the ubiquitin/proteasomal and lysosomal pathways are known to participate in ErbB receptor family degradation in response to ligand stimulation [32,33]. We next evaluated the effects of a proteasomal inhibitor (MG132) and a lysosomal inhibitor (bafilomycin A1) on EGF-mediated downregulation of EGFR in A549 cells. As shown in Figure 5A, pretreatment with MG132 only slightly reversed EGFR degradation caused by 10 min of EGF treatment. In contrast, blocking of lysosomal degradation by bafilomycin A1 dramatically reversed EGFR downregulation caused by EGF treatment at several time points (10 min, 30 min, 1 h, and 24 h) (Figure 5B), suggesting that EGF-mediated EGFR degradation mainly occurs through a lysosomal degradation pathway. In the EGF-induced lysosomal degradation pathway, EGFR is first internalized and is then transported to lysosomes for degradation; thus, endocytosis is required for degradation [34]. Therefore, we first tested whether IL-17A affects the endocytosis of EGFR. A549 cells treated with vehicle, EGF, or EGF+rhIL-17A for 10 or 30 min were stained with a PE-conjugated EGFR monoclonal antibody (mAb) or a PE-labeled isotype-matched mAb, and the intensity of membranous EGFR was analyzed by flow cytometry. Our results showed that the EGFR intensity was indistinguishable in cells treated with EGF alone or EGF with IL-17A (Figure 5C), suggesting that IL-17A did not affect EGF-induced EGFR endocytosis. We further analyzed the effect of IL-17A on EGF-induced transport of EGFR to lysosomes using the specific lysosomal marker, LAMP1. As shown in confocal microscopy images (Figure 5D, left panel), an increase in the co-localization of EGFR with LAMP1 (as indicated by a white arrow) induced by 30 min of treatment with EGF was significantly reversed by IL-17A cotreatment (Figure 5D, right panel), implying that IL-17A may impair EGFR lysosomal degradation caused by EGF. In addition to the colocalization of EGFR and lysosomes, we also observed that EGF-induced EGFR nuclear localization (orange arrow indicated) increased in the presence of IL-17A (Figure 5D, right panel).

## 4. Discussion

Over the past decade, multiple lines of evidence have suggested that IL-17A/Th17 may play an oncogenic role in lung cancer. For example, high IL-17A levels were reported to be correlated with increased lymph node invasion and distant metastases in NSCLC [35]. IL-17A was shown to induce the production of vascular endothelial growth factor (VEGF)-A, VEGF-C, and VEGF-D that correlated with increased angiogenesis and lymphangiogenesis in NSCLC [23,36]. Moreover, IL-17A can induce increases of migration, invasion, and stemness in NSCLC cells such as A549, H460, and H1975 cells via p38 mitogen-activated protein kinase (MAPK) and STAT3/nuclear factor (NF)-κB/Notch1 signaling [20,21]. In addition to the critical role of IL-17A in regulating metastasis of NSCLC, some evidence has supported the proliferative role of IL-17A in cancer such as multiple myelomas [37,38]. As we know from previous studies, EGFR activation plays an important role in promoting proliferation of NSCLC cells [39]. This study aimed to investigate the impacts of IL-17A on proliferation of NSCLC cells and the crosstalk between IL-17A and EGFR activation.

EGFR is an important transmembrane glycoprotein that is expressed in organs with epithelial cells, such as the lungs, skin, and gastrointestinal tract. In the HCC827 and PC9 EGFR-mutant NSCLC cell lines, we found that overexpression of IL-17A or treatment with rhIL-17A both enhanced EGFR activation, cell proliferation, and EGFR-TKI resistance. Chen et al. reported that IL-17A can induce keratinocyte proliferation and skin tumor formation via activating EGFR. Mechanistically, IL-17R interacts with EGFR via the adaptor proteins, tumor necrosis factor (TNF) receptor-associated factor 4 (TRAF4) and Act1, to further recruit c-Src for IL-17A-induced EGFR transactivation [18,40]. Src kinase was shown to play an important role in transactivation of EGFR without EGF stimulation [41]. In our study, we found that IL-17RC depletion abrogated rhIL-17A-induced EGFR activation, suggesting that IL-17A-enhanced EGFR activation is dependent on IL-17R in EGFR-mutant NSCLC cells. Moreover, we also observed that overexpression of IL-17A in HCC827 cells and treatment of rhIL-17A in PC9 cells both induced Src phosphorylation (Figure S7). Hence, this suggests that IL-17A might induce the formation of an IL-17R-EGFR-TRAF4-Act1 complex to further recruit Src, resulting in EGFR activation in EGFR-mutant NSCLC cells, and this possibility should be investigated in the future. In addition to EGFR activation triggered by the IL-17A-IL17RC axis, our RTK array data showed that phosphorylation of other RTKs including ErbB2, ErbB3, RYK, DDR2, EphA10, and HGFR/c-Met was upregulated in rhIL-17A-treated PC9 cells. Our study showed that both IL-17A overexpression and rhIL-17A treatment induced resistance to the pan-ErbB-TKI, afatinib, in EGFR-mutant NSCLC cells. Amplification and activation of c-Met are recognized as important bypass pathways involved in resistance to afatinib [16]. Whether Met activation is required for IL-17A-mediated afatinib resistance was further investigated in our study. Actually, SU11274, a c-MET inhibitor, increased the sensitivity of rhIL-17A-treated PC9 cells to afatinib, suggesting that IL-17A may activate compensatory EGFR signaling via phosphorylating c-Met under afatinib treatment in EGFR-mutant NSCLC cells. In addition to c-Met, other possible RTKs induced by IL-17A were also reported to affect the sensitivity of EGFR-TKIs in NSCLC such as RYK [42]. Whether RYK plays a role in IL-17A-mediated EGFR-TKI resistance needs to be further investigated.

In NSCLC cells with WT-EGFR, our results from kinase array and Western blotting analyses showed that the IL-17A-IL-17RC axis enhanced EGF-induced activation of EGFR and its downstream signals including Akt and Stat3. Moreover, EGF-induced cell proliferation was also accelerated by rhIL-17A cotreatment in A549 cells, suggesting that the IL-17A-IL-17RC axis may cooperate with the EGF-EGFR axis to promote proliferation of WT-EGFR NSCLC cells. EGF treatment of NSCLC cells leads to EGFR degradation through receptor-mediated endocytosis and endosomal trafficking to lysosomes [43]. We actually observed that total EGFR was downregulated after EGF treatment in A549 and H23 cells. Interestingly, EGF-mediated downregulation of EGFR was partially reversed by the presence of IL-17A in WT-EGFR NSCLC cells, but this phenomenon disappeared when IL-17RC was knocked down. These results suggest that the IL-17A-IL-17RC axis may attenuate EGF-mediated EGFR degradation to further enhance EGFR activation and proliferation caused by EGF. We further investigated how IL-17A affects EGF-mediated EGFR degradation. According to data from flow cytometry, we found the EGF-mediated endocytosis was not influenced by the presence of IL-17A. In contrast, from confocal microscopy, we observed that the EGF-induced increase in the colocalization of EGFR and lysosomes was significantly reversed by the presence of IL-17A, suggesting that IL-17A may influence the trafficking of EGFR to lysosomes and further attenuate EGF-induced lysosomal EGFR degradation. However, how IL-17A affects the trafficking mechanisms of EGFR from endosomes to lysosomes needs to be further investigated in the future.

In addition to EGF-induced EGFR degradation, accumulating evidence indicates that EGFR is internalized and transported to nuclei rather than being degraded or recycled back to the cell surface. Nuclear EGFR was strongly correlated with poor OS in several cancer types, was associated with drug resistance, and was found to be involved in regulating genes needed for cell proliferation [44]. Actually, IL-17E shares a common co-receptor with IL-17A and was shown to synergize the EGF-induced EGFR nuclear translocation and contribute to EGFR-TKI resistance [19]. From results of confocal microscopy, we also observed that EGF-induced EGFR nuclear localization increased in the presence of IL-17A in A549 cells, suggesting that this might be an another action of IL-17A causing enhancement of EGF-induced proliferation in NSCLC cells harboring WT-EGFR.

## 5. Conclusions

In summary, our results indicated the diverse impacts of the IL-17A/IL-17RC axis on EGFR activation in NSCLC cells with WT and mutant EGFR. In NSCLC cells with mutant EGFR, we found that the IL-17A/IL-17RC axis enhanced phosphorylation of EGFR and Met, thereby promoting proliferation and resistance to EGFR-TKIs. In NSCLC cells with WT EGFR, the IL-17A/IL-17RC axis promoted EGF-induced EGFR activation and cell proliferation by causing impairment of the trafficking of EGFR to lysosomes. We suggest that the presence of IL-17A within the tumor microenvironment of NSCLC probably promotes and sustains EGFR activation and ultimately results in tumor proliferation. Therefore, therapeutic strategies that target IL-17A or its receptor could represent an effective method for NSCLC treatment.

## Data Availability

All data used during the current study are available from the corresponding author upon reasonable request.

## Supplementary Materials

The following supporting information can be downloaded at https://www.mdpi.com/article/10.3390/cancers15133288/s1. Figure S1: A Kaplan–Meier (KM) plotter database was used to evaluate the correlation between interleukin (IL)-17RA expression levels and overall survival (OS) in patients with lung cancer from a GEO (GSE50081) or TCGA dataset; Figure S2: Overexpression of interleukin (IL)-17A facilitates phosphorylation of epidermal growth factor receptor (EGFR) and Met in human non-small-cell lung cancer (NSCLC) cells harboring different EGFR mutations; Figure S3: Effect of recombinant human interleukin (IL)-17A (rhIL-17A) treatment on phosphorylation of epidermal growth factor receptor (EGFR) and Met in human non-small-cell lung cancer (NSCLC) cells; Figure S4: RT-qPCR was performed to detect interleukin-17 receptor C (IL-17RC) expressions in PC9 and A549 cells after transducing IL-17RC short hairpin (sh)RNA or control shRNA (shLuc); Figure S5: Effect of interleukin (IL)-17A on the half maximal inhibitory concentration (IC50) value of afatinib in human non-small-cell lung cancer (NSCLC) cells. Figure S6: Detection of phosphorylated epidermal growth factor (EGF) receptor (p-EGFR) and total EGFR by Western blotting after treatment of H23 cells with 100 ng/mL EGF or EGF+10 ng/mL recombinant human interleukin (rhIL)-17A for 10 min; Figure S7: Detection of phosphorylated (p)-Src by Western blotting in HCC827 and PC9 cells which were, respectively, transfected with interleukin (IL)-17-His and treated with recombinant human (rh)IL-17A (0, 10, and 50 ng/mL) for 24 h.
