# Supplementary material for: Sustaining the Activation of EGFR Signal by Inflammatory Cytokine IL17A Prompts Cell Proliferation and EGFR-TKI Resistance in Lung Cancer
Source: Cancers (Basel). 2023 Jun 22;15(13):3288. doi: 10.3390/cancers15133288 (PMC10340054; doi:10.3390/cancers15133288)
Supplement: Supplementary file 1 [file cancers-15-03288-s001.zip › cancers-2423817-supplementary.pdf]

cancers

MDPI

Article

Sustaining the activation of EGFR signal by inflammatory cytokine IL17A prompts cell proliferation and EGFR-TKI resistance in lung cancer

Kai-Ling Lee, Tsung-Ching Lai, Wei-Jiunn Lee, Yu-Chieh Chen, Kuo-Hao Ho, Wen-Yueh Hung, Yi-Chieh Yang, Ming-Hsien Chan, Feng-Koo Hsieh, Chi-Li Chung, Jer-Hwa Chang, and Ming-Hsien Chien

Supplementary materials and methods

Methods

Real-Time Quantitative Polymerase Chain Reaction (RT-qPCR)

Cellular messenger (m)RNA was isolated with the TRIzol reagent (Invitrogen Life Technologies, Carlsbad, CA, USA). To obtain complementary (c)DNA, 5 µg of total RNA was reverse-transcribed using iScript™ Reverse Transcription Supermix (Bio-Rad Laboratories, Irvine, CA, USA) according to the manufacturer's instructions. The qPCR was performed on a real-time PCR system (ABI 7300) using SYBR Green Master Mix (Bio-Rad Laboratories). Primer sequences used in this study were as follows: IL17RC forward: CTGGGAAGAGCCTGAAGATG and reverse: CTTCTCGTACCTGGGCTGAG; GAPDH forward: CTATAAATTGAGCCCGCAGC and reverse: ATGACAAGCTTCCCGTTCTC.

Supplementary data

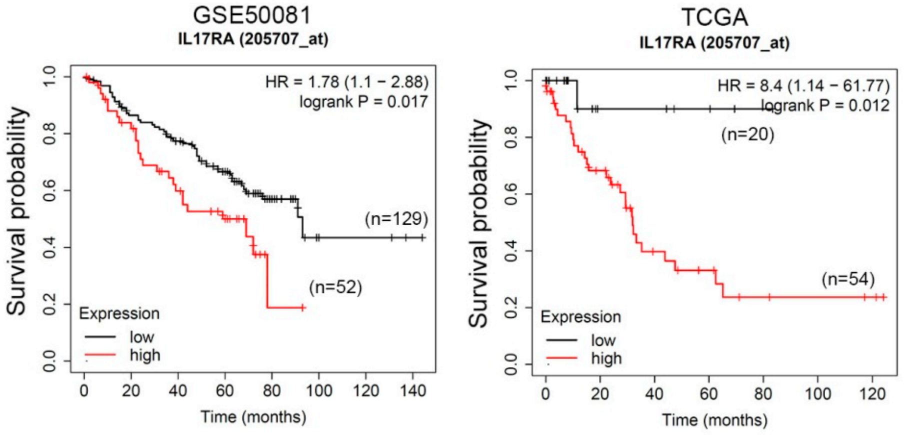

Supplementary Figure S1. A Kaplan-Meier (KM) plotter database was used to evaluate the correlation between interleukin (IL)-17RA expression levels and overall survival (OS) in patients with lung cancer from a GEO (GSE50081) or TCGA dataset. Gene expression was dichotomized into high and low values using the best cut-off. *P*<0.05 was considered to indicate a statistically significant difference. HR, hazard ratio.

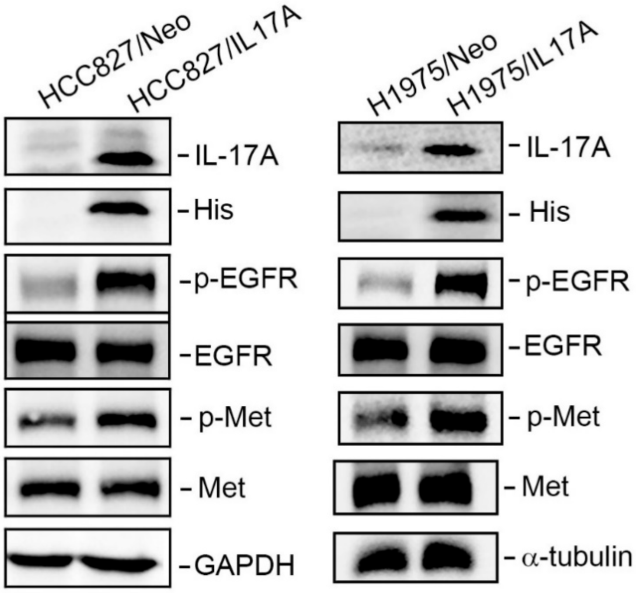

Supplementary Figure S2. Overexpression of interleukin (IL)-17A facilitates phosphorylation of epidermal growth factor receptor (EGFR) and Met in human non-small cell lung cancer (NSCLC) cells harboring different EGFR mutations. NSCLC cells with different EGFR mutations including HCC827 (exon 19 deletion) and H1975 (L858R and T790M) cells were transfected with control vector (Neo) or IL-17A-expressing vector (IL-17A-His) and further detected levels of phosphorylated EGFR and Met by a Western blot analysis.

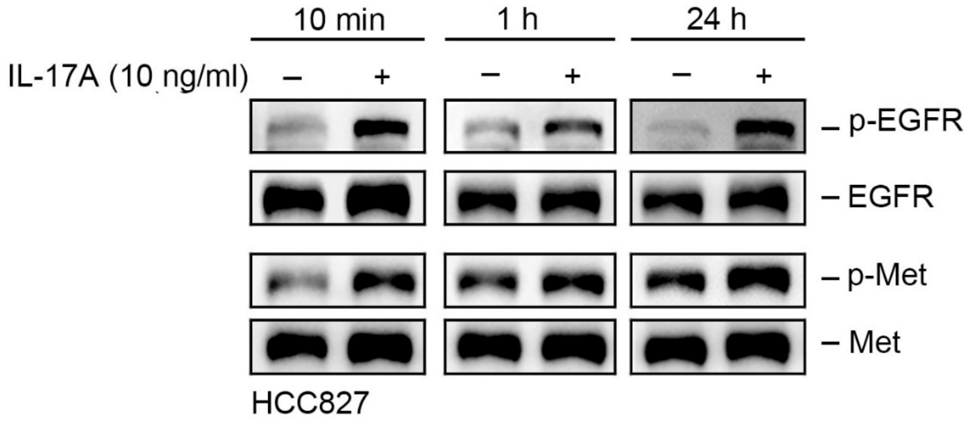

Supplementary Figure S3. Effect of recombinant human interleukin (IL)-17A (rhIL-17A) treatment on phosphorylation of epidermal growth factor receptor (EGFR) and Met in human non-small cell lung cancer (NSCLC) cells. HCC827 cells were treated with vehicle or 10 ng/ml rhIL-17A for indicated time points (10 min, 1h, and 24 h) and a Western blot analysis was used to detect expression levels of phosphorylated EGFR and Met.

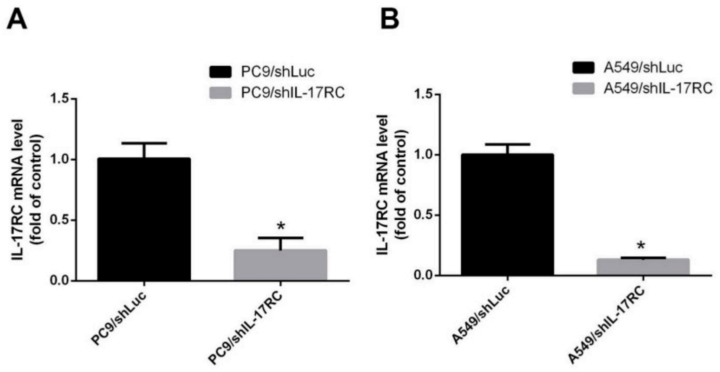

Supplementary Figure S4. RT-qPCR was performed to detect interleukin-17 receptor C (IL-17RC) expressions in PC9 and A549 cells after transducing IL-17RC short hairpin (sh)RNA or control shRNA (shLuc). Quantitative results of IL-17RC mRNA levels were adjusted to GAPDH mRNA levels. **P* < 0.05, compared to the vehicle group.

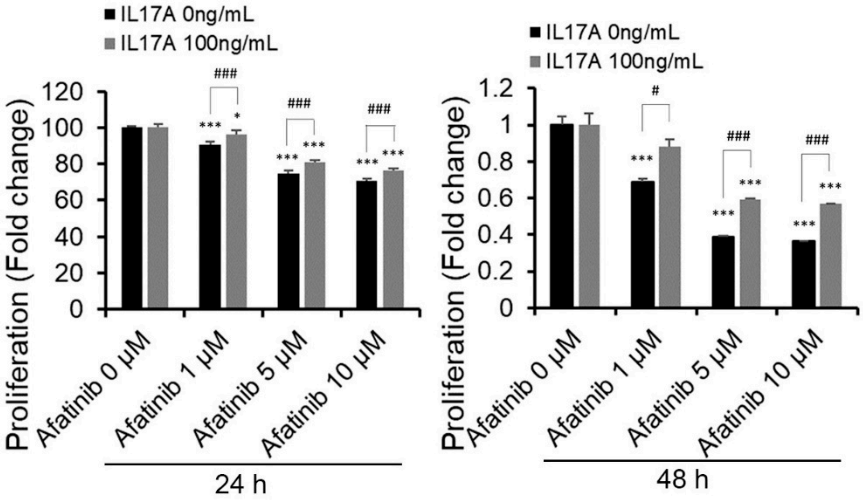

Treatment	Time (h)	IC50 (μM)
Vehicle	24	>10
rhIL-17A	24	>10
Vehicle	48	3.52±0.09
rhIL-17A	48	>10

Supplementary Figure S5. Effect of interleukin (IL)-17A on the half maximal inhibitory concentration (IC50) value of afatinib in human non-small cell lung cancer (NSCLC) cells. PC9 cells were treated with different concentrations of afatinib for either 24 h or 48 h, in the presence or absence of 100 ng/ml of recombinant human interleukin (rhIL)-17A. The IC50 values of these cells were then determined using a CCK8 assay.

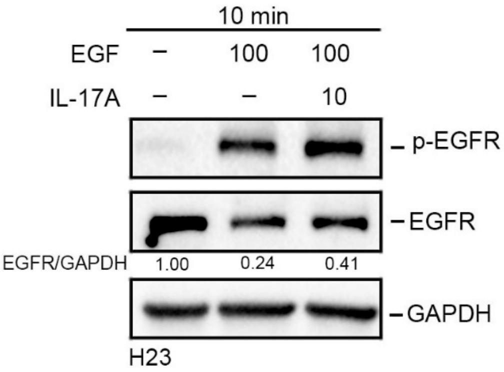

Supplementary Figure S6. Detection of phosphorylated epidermal growth factor (EGF) receptor (p-EGFR) and total EGFR by Western blotting after treatment of H23 cells with 100 ng/ml EGF or EGF+10 ng/ml recombinant human interleukin (rhIL)-17A for 10 min. Quantitative results of total EGFR proteins were adjusted to GAPDH protein levels.

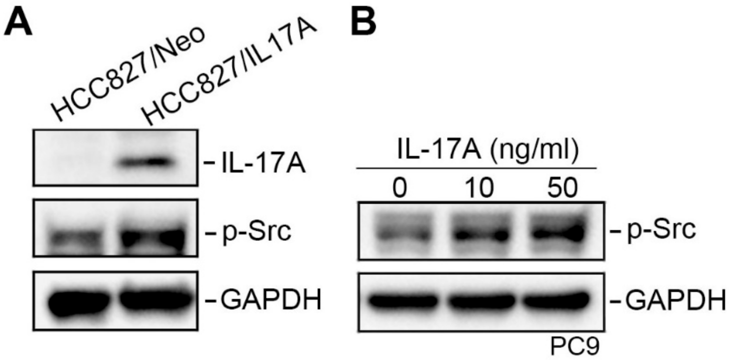

Supplementary Figure S7. Detection of phosphorylated (p)-Src by Western blotting in HCC827 and PC9 cells which were respectively transfected with interleukin (IL)-17-His and treated with recombinant human (rh)IL-17A (0, 10, and 50 ng/ml) for 24 h. Quantitative results of p-Src proteins were adjusted to GAPDH protein levels.
